# Fine-Scale Patterns of Genetic Structure in the Host Plant *Chamaecrista fasciculata* (Fabaceae) and Its Nodulating Rhizobia Symbionts

**DOI:** 10.3390/plants9121719

**Published:** 2020-12-07

**Authors:** Mahboubeh Hosseinalizadeh Nobarinezhad, Lisa E. Wallace

**Affiliations:** 1 Department of Biological Sciences, Mississippi State University, Starkville, MS 39762, USA; 2 Department of Biological Sciences, Old Dominion University, Norfolk, VA 23529, USA; lewallac@odu.edu

**Keywords:** 16S rRNA, *Chamaecrista fasciculata*, fine-scale spatial genetic structure, microsatellite, rhizobia, *truA*

## Abstract

In natural plant populations, a fine-scale spatial genetic structure (SGS) can result from limited gene flow, selection pressures or spatial autocorrelation. However, limited gene flow is considered the predominant determinant in the establishment of SGS. With limited dispersal ability of bacterial cells in soil and host influence on their variety and abundance, spatial autocorrelation of bacterial communities associated with plants is expected. For this study, we collected genetic data from legume host plants, *Chamaecrista fasciculata,* their *Bradyrhizobium* symbionts and rhizosphere free-living bacteria at a small spatial scale to evaluate the extent to which symbiotic partners will have similar SGS and to understand how plant hosts choose among nodulating symbionts. We found SGS across all sampled plants for both the host plants and nodulating rhizobia, suggesting that both organisms are influenced by similar mechanisms structuring genetic diversity or shared habitat preferences by both plants and microbes. We also found that plant genetic identity and geographic distance might serve as predictors of nodulating rhizobia genetic identity. *Bradyrhizobium elkanii* was the only type of rhizobia found in nodules, which suggests some level of selection by the host plant.

## 1. Introduction

Several studies have recognized plants as one of the most important factors shaping soil microbial community structure [1,2,3,4]. It has been suggested that this may be the result of active selection by plants for certain soil microbes or shared habitat preferences by plants and microbes [5] because spatially close biological communities are more similar than expected by chance and this similarity decays with distance [6]. It has also been demonstrated that differences in microbial community composition are significantly correlated with phylogenetic distance of their plant hosts [7]. Plant species that develop specific associations with soil microbes have important implications for coexistence in natural communities [5], and recent evidence suggests that microbial communities are influenced by both dispersal limitation and host plants [8]. In ref. [9], by studying plant phylogeny and life history of summer annuals in an agricultural field, it was indicated that rhizosphere beta-diversity was positively correlated with phylogenetic distance between plant species, but not genetic distance within a plant species. Plants within a closely related taxonomic group are likely to share traits, including amount and availability of rhizodeposits and root defensive strategies, and these are key factors shaping soil microbial communities [10]. Thus, plant hosts with similar genotypes may have similar soil microbial communities [1,11,12] which may indicate the presence of genotype x genotype interactions in these situations.

While most studies have focused on differences in soil microbial communities associated with different plant species, a few studies have addressed these differences within a single species and found that microbial community structure and composition can also vary according to intraspecific genotypic differences of host plants [1,13,14,15]. These studies have demonstrated that diversity in community structure of the rhizosphere microbiome can be partially explained by the genotype of their plant hosts. For example, ref. [16] demonstrated that the host genotype accounts for approximately 5.7% of the variance in the *Hordeum vulgare* L. rhizosphere microbiome composition. Therefore, with dispersal-limited bacterial and fungal communities and host influence on microbial variety and abundance, spatial autocorrelation of rhizosphere communities is expected [6,17]. However, the underlying mechanisms by which the plants drive the rhizosphere microbiome are not well understood. Thus, variation in the rhizosphere microbiome across diverse plant species and natural systems should be subjected to more research.

Nodulating rhizobia, which begin as free-living soil microbes, also experience variable selection as a result of changes in host genetic identity, resource availability, temperature, moisture and the soil biotic community. Both interspecific [18,19] and intraspecific [20,21,22] genetic variations of hosts influence selection on nodulating rhizobia. Even variation at a single host gene can have dramatic effects on strain occupancy and fitness in nodules [23] and on the frequency of microbial genera in the rhizosphere [24]. The plant effects often reciprocally depend on the rhizobial lineage and can be a result of genetic variation in a single rhizobial gene [25]. Numerous studies have documented that legume–rhizobial symbioses show a high level of specificity, occurring at both species and genotypic levels [26,27]. It is also shown that the bacterial diversity in total nodules of a host plant is significantly lower than that in the corresponding rhizosphere [28], which confirms the presence of selection by plant hosts to associate with certain types of rhizobia in the soil. Thus, it is expected that the presence of significant genotype x genotype interactions between plants and their symbionts would reinforce this pattern at fine spatial scales. Therefore, analyzing the genetic structures of host plants and their symbionts is helpful to better understand the functional spatial scale of such interactions.

*Chamaecrista fasciculata* (Michx.) Greene (Partridge Pea), an annual native legume (Fabaceae), is widely distributed in the eastern U.S. The species exhibits intraspecific morphological variation and wide ecological tolerance of different soil types and habitats [29]. As a species capable of harboring nitrogen-fixing rhizobia, it is an important species in many natural ecosystems because it provides nitrogen for other plants, as well as cover, nectar, and pollen for animals. It has also been of interest in agricultural systems, for example in crop rotation to enhance soil nitrogen [30] and to manage root-knot nematodes [31]. Given its annual habitat, herbaceous growth form, and phylogenetic position as a nodulating form outside of the Papilionoid clade, there is growing interest in developing the Partridge Pea as a model for studies of legume evolution [32]. *Bradyrhizobium* is the only genus of rhizobia that is symbiotic with *C. fasciculata*, but many different genetic variants of *Bradyrhizobium* are able to nodulate in *C. fasciculata* [33,34,35,36].

The findings of ref. [36] indicated that in Mississippi, *Bradyrhizobium* are phylogenetically diverse within a site but do not have a strong genetic structure across large geographic distances. This led them to suggest that the scale at which successful symbioses are established may be very fine and determined by plant x rhizobia genotypic interactions, rather than soil traits. In this study, we tested for the presence of similar patterns of fine-scale genetic structure between *C. fasciculata* host plants and their nodulating *Bradyrhizobium* symbionts. We expected that plants in close physical proximity would be more genetically similar than those more distant from one another due to having gravity dispersed seeds. We also expected that nodulating rhizobia would exhibit a spatial scale of genetic structure that is concordant with their host plants due to genotype x genotype interactions, limited dispersal of bacteria in the soil, and enhancement of a suitable symbiont pool from continual host plant presence. Lastly, we also characterized members of order Rhizobiales, which includes *Bradyrhizobium*, from plant rhizospheres to test the hypothesis that the bacterial strains in the nodules are a subset of available bacteria in the soil. Previous studies have surveyed genetic diversity and distributions of rhizobia communities in the soil and nodules of different plant hosts [24,37,38,39], but this study covers the subject at a different spatial scale. This fine-scale study directly tests for an association between genotypic diversity of host plants and their *Bradyrhizobium* symbionts and provides understanding of the degree to which host plants choose certain microbial genotypes from the soil. Additionally, examination of fine-scale genetic structure of multiple interacting species has rarely been conducted. The proposed study enhances our understanding of the role of bacterial symbionts in local adaptation of plants and the degree to which plant hosts act as a predictor to determine genetic structure of soil microbes.

## 2. Results

No clonal genotypes were detected in the plant dataset. The number of microsatellite alleles per locus ranged from 3 to 12, with a mean value of 6.78 across all plants (Table 1). The percentage of polymorphic loci (%P) was 100 in each of the three plots, and the observed and expected heterozygosity ranged from 0.14 to 0.71 and 0.32 to 0.84, respectively. The inbreeding coefficients (F_IS_) were consistently positive, indicating heterozygote deficiency across all plots (Table 1).

**Figure 1:**
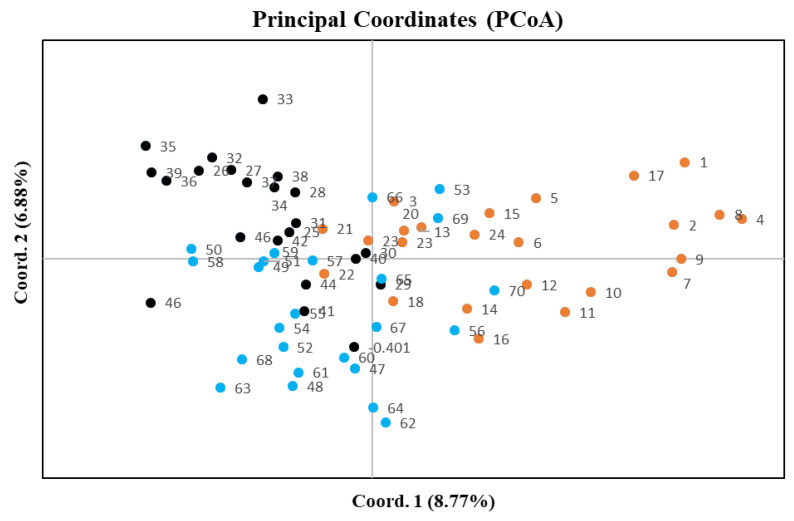
A principle coordinates analysis (PCoA) of sampled plants based on microsatellite variation in all three plots. Orange circles = plot 1, black circles = plot 2, blue circles = plot 3. First and the second components account for 8.77% and 6.88% of variation in the dataset, respectively.

**Figure 2:**
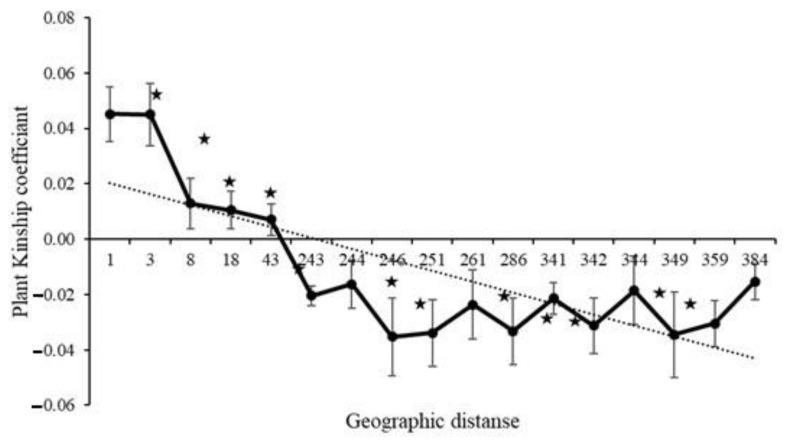
Ritland’s kinship coefficients for pairs of plants plotted against the logarithm of geographic distance between pairs and the estimated regression line (regression slope bF = −0.01214, *p* = 0.000). Significant autocorrelation at a distance class is indicated by a star.

Among the three plots only plot 1 demonstrated a significant regression slope (b_F_) for plant kinship coefficient over distance classes (*p* = 0.001 for b_F_ = −0.0138) (Table 2). Since the genetic diversity measures among all three plots were very similar (Table 1) and principle coordinate analysis did not separate individuals by plot (Figure 1), we combined samples from the plots for downstream analyses. When considering the combined plots, we found significant autocorrelation in plant genotypes for all distance classes except 244, 261, 344 and 384 m, which are distributed at the intermediate and largest distances (Figure 2). The average kinship coefficient decreased linearly with the natural logarithm of spatial distance (r_ij_) between individuals. Significant positive values of F_ij_ were found at short distances (<43 m), indicating that neighboring individuals had a higher genetic relatedness than random pairs of individuals. Negative values (i.e., individuals within a distance class were less genetically similar than expected with a random distribution) of F_ij_ occurred at larger distances (Figure 2). Mean values of the regression slope (b_F_), F_(1)_ and Sp statistics across all plots were −0.0121, 0.045 and 0.0127, respectively (Table 2). The *p*-value for the regression slope was 0.000.

**Figure 3:**
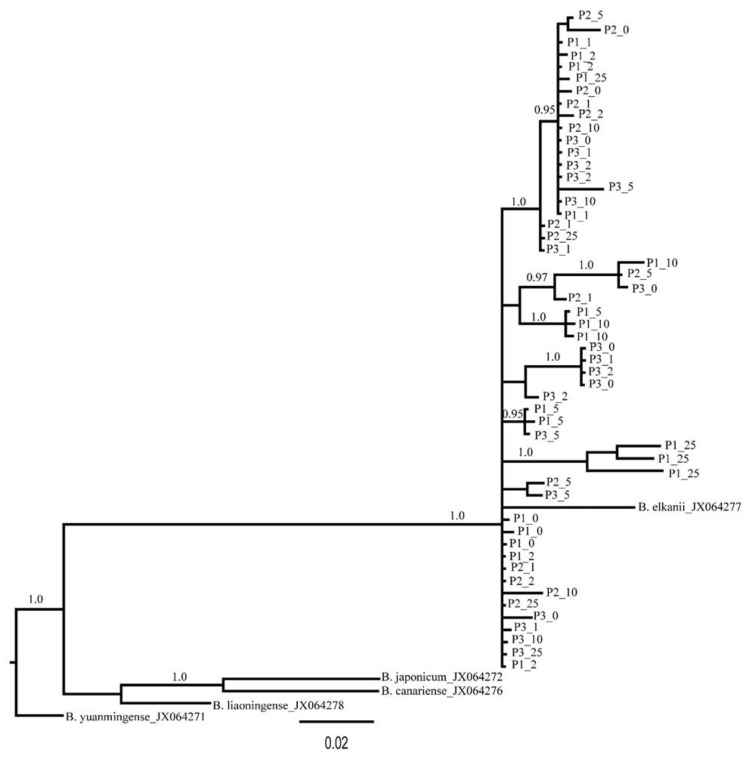
Phylogenetic tree of nodualting *Bradyrhizobium* based on *truA* sequences. The first and second numbers of each tip indicate plot number (1, 2 or 3) and distance interval (0, 1, 2, 5, 10 or 25). Values on the branches are posterior probability (PP). Support of less than 0.95 PP is not shown.

**Figure 4:**
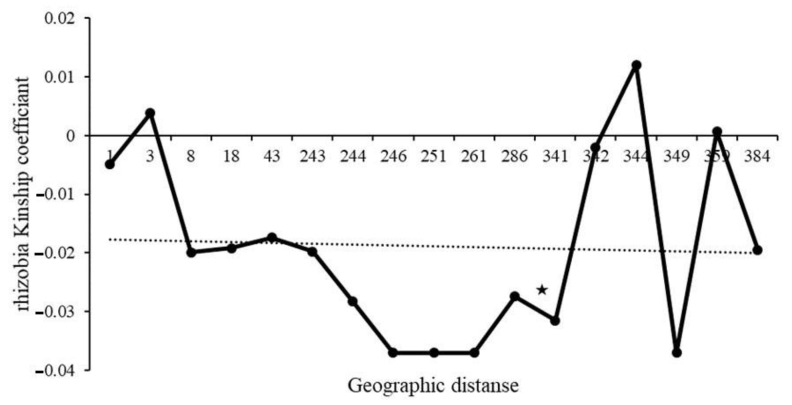
Ritland’s kinship coefficients for pairs of nodulating Bradyrhizobium plotted against the logarithm of geographic distance separating members of the pairs. A significant autocorrelation, shown with a star, was only found for distance class 286. regression slope bF = −0.0031, *p* = 0.041.

Due to the lack of mature nodules for some of the collected plants, we failed to culture rhizobia for five plants, and sequencing was unsuccessful for 12 samples of the cultured strains. Thus, we were able to generate 53 *truA* sequences from nodulating rhizobia. The aligned length of the *truA* sequence set was 497 nucleotides (~67% coverage of *truA* gene in *Bradyrhizobium*). All sequences most closely resembled *Bradyrhizobium elkanii truA* sequences in BLAST-n searches in GenBank (NCBI 1988). Additionally, the phylogeny of *truA* sequences indicates that all recovered sequences from *C. fasciculata* cluster exclusively with *B. elkanii* and with 1.0 posterior probability support (Figure 3). The sequence diversity of the nodulating rhizobia was found to be similar in all three plots. A similar number of haplotypes (6–9) was found across the plots, and these haplotypes exhibited low mean genetic difference between pairs of sequences (π = 0.03457–0.04175; Table 3). Across the three plots 23 distinct rhizobia *truA* haplotypes were found. Only plot 1 demonstrated a significant regression slope (b_F_) for rhizobia kinship coefficient over distance classes (b_F_ = −0.0165, *p* = 0.046) (Table 2). When plots were combined, we found no significant autocorrelations in rhizobia genotypes for any distance classes except for 286 m (Figure 4), but we found a significant regression slope for rhizobia kinship coefficients against distance among pairs of individuals (b_F_ = −0.0031, *p* = 0.041) (Table 2).

**Figure 5:**
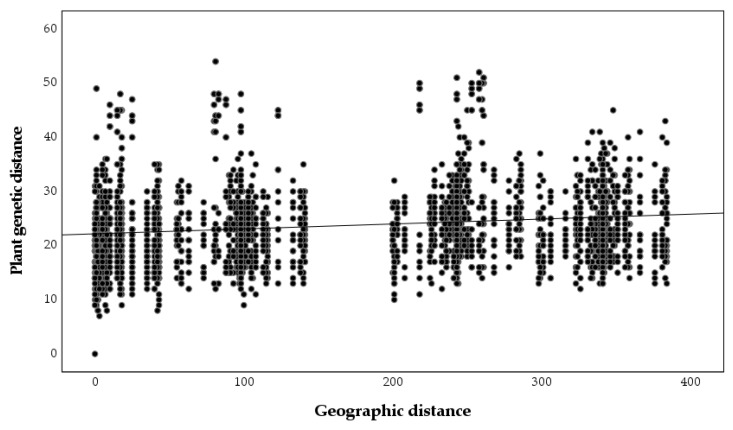
Pairwise plant genetic distances based on microsatellite alleles compared to pairwise geographic distance between sampled plants (r = 0.243, *p* = 0.001).

**Figure 6:**
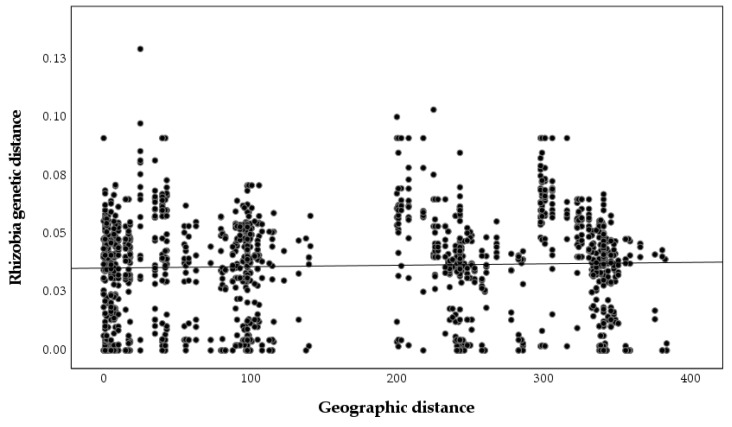
Pairwise nodulating *Bradyrhizobium* genetic distances based on *truA* sequences compared to pairwise geographic distance between sampled nodules (r = 0, *p* = 0.99).

**Figure 7:**
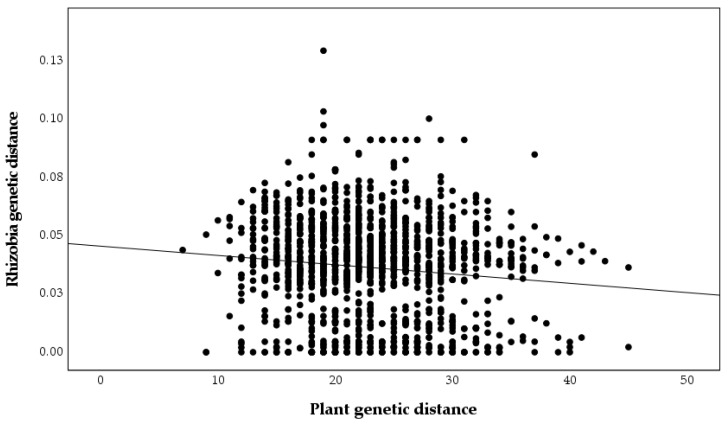
Pairwise plant genetic distances based on microsatellite alleles compared to pairwise nodulating *Bradyrhizobium* genetic distances based on *truA* sequences (r = −0.087, *p* = 0.261).

A significant relationship between plant genetic and geographic distances was found (r = 0.243, *p* = 0.001, Figure 5), but the relationship between nodulating rhizobia genetic and geographic distances was not significant (r = 0, *p* = 0.99, Figure 6), as was the relationship between plant genetic and nodulating rhizobia genetic distance (r = −0.087, *p* = 0.26, Figure 7). Stepwise regression analysis showed that plant genetic distance, and plant genetic plus geographic distance, are both able to predict genetic distance of nodulating rhizobia with the adjusted r-square of 0.012 and 0.016, respectively (*p* < 0.0001 for both, Table 4). Geographic distance alone did not significantly determine nodulating rhizobia genetic distance. Even though they were significant, the combined variables of plant genetic distance and geographic distance only explained 1.6% of the observed variation in nodulating rhizobia genetic distances (Table 4).

**Figure 8:**
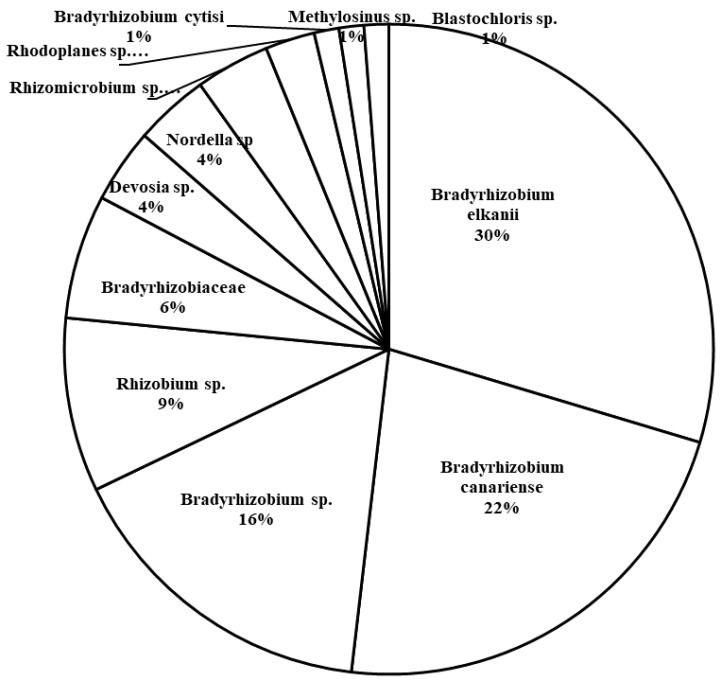
Abundance of families and genera of Rhizobiales found in rhizosphere samples of *Chamaecrista fasciculata* in the current study.

The total number of unique 16S rRNA sequences of Rhizobiales present in the rhizosphere samples from host plants in all three plots was 81 sequences. The majority of the retrieved sequences are of *Bradyrhizobium*, including *B. elkanii* (30%), *B. canariense* (22%), and unidentified *Bradyrhizobium* strains (9%) (Figure 8). Rhizobium strains were second in abundance, making up 9% of the rhizosphere sequences.

## 3. Discussion

In many plant species, seed dispersal has a peak distribution at or close to the maternal plant and progressively fewer seeds are dispersed at greater distances from the maternal plant [40,41,42]. The extent of SGS within plant populations depends on factors such as seed and pollen dispersal distance and effective plant density [43]. According to the theory of isolation by distance, SGS arises from the interplay of limited gene flow and local genetic drift, and the rate of decrease of genetic similarity with distance is a measure of the strength of SGS [44,45,46]. The morphological characteristics of *C. fasciculata*, including heaviness of the seeds and bee pollination, suggest that plants should exhibit an isolation by distance pattern. The SGS pattern detected for host plants in the studied plots was consistent with IBD, showing an increase in genetic distance between pairs of plants as they increased in physical distance from one another. Additionally, the kinship coefficients gradually decrease from very positive to very negative over the distance of sampling. Discovering statistically significant fine scale genetic structure at most of our studied distances is concordant with the existence of very restricted seed dispersal events among plants and suggests that plants are not randomly distributed even at this fine scale. Values for the Sp statistic in this study, ranging 0.0048–0.0139, are comparable to another study on this species with reported Sp statistic of 0.00746 [47]. The mean Sp statistic in other outcrossing or gravity dispersed plant species is 0.0126 and 0.0281, respectively [43], which are comparatively close to our measured Sp statistic value for *C. fasciculata*.

Given the presence of fine-scale structure in host plants and widely documented genotype x genotype interactions between legumes and their symbionts, we expected to find significant genetic structure in nodulating rhizobia of *C. fasciculata* as well. Additionally, limited dispersal ability of bacterial cells in the soil due to passive means is expected to maintain structure if there is not extensive soil disturbance. Although we did not find a significant kinship coefficient for pairs of rhizobia at most geographic distances, the mean sp statistic in rhizobia followed the same pattern observed for plant hosts. That is, we found a significant b_F_ for rhizobia and plant hosts in plot 1 and the combined plots. This finding suggests a common mechanism that influences genetic structure of plants and rhizobia. It may be that host plants directly influence their rhizobia symbionts by selecting for certain strains or that plant hosts and their nodulating rhizobia are influenced by similar environmental factors that structure genetic diversity. In our study, we focused on only one housekeeping gene, *truA*, to identify the nodulating rhizobia and estimate genetic diversity of this community. Although this does not capture genome-wide diversity, it is a suitable genetic marker that was considered highly informative of phylogenetic relationships in *Bradyrhizobium* by refs. [36,48]. Our study revealed relatively high haplotype diversity (Hd = 0.82–0.86 across plots) in *truA*, which indicates an ability to discriminate among most of the rhizobia symbionts we recovered. In another study, the nucleotide diversity of *Rhizobium leguminosarum* biovar *viciae* isolates from *Vicia cracca* L. acquired using two housekeeping genes, *glnII* and *recA*, and one nodulation gene, *nodC*, revealed relatively close values of nucleotide diversity for all of the three studied genes (π = 0.036, 0.021 and 0.025, respectively) [49]. This study suggests that single genes can be informative of bacterial genetic diversity.

While a literature review found no fine-scale studies of genetic structure in nodulating rhizobia or plant endophytes for comparison to our results, we have found that the number of haplotypes recovered for *C. fasciculata* is similar to studies of other legumes. In ref. [36], which also used *truA* to study genetic diversity of nodulating rhizobia in *C. fasciculata* across Mississippi, 9–25 haplotypes and 0.831–0.954 for haplotype diversity was reported at a regional scale. Our finding of six haplotypes and substantial haplotype diversity in a single site indicates that populations can harbor diverse symbionts even at the smallest spatial scales. In a fine scale study (<2 m) of diversity patterns in microbial communities associated with roots of *Bistorta vivipara* L., a perennial herb, it was revealed that similarities between the structure of bacterial and fungal communities were stronger in soils than in roots [50]. The root-associated fungal and bacterial communities in that study both showed significant spatial autocorrelation at distances below 40–50 cm, while soil-associated communities showed no spatial structure across the 2 m scale investigated. This result, along with ours, suggests that different host filters are responsible for structuring symbiotic endophytes of roots and nodules.

Host promiscuity in symbiotic associations can influence exotic legume establishment and colonization of novel ranges [51], and it has been suggested that enhanced diversity of symbionts may underlie the success of host plants in non-native habitats (e.g., [52]). Hence, it may be expected that due to a broad geographic distribution of a host species it potentially harbors different rhizobia strains that enable tolerance to varying soil types and environmental conditions. Unlike this assumption and the previous study on this system, which covered a vast geographic area with different physiographic regions and found different genetic variants of *Bradyrhizobium* nodulating *C. fasciculata* [36], in our 400 m study area, the only sequences retrieved from nodules of the widespread *C. fasciculata* studied here belonged to *Bradyrhizobium elkanii* (Figure 3). While our results confirm the exclusive use of this *Bradyrhizobium* by *C. fasciculata* also noted in other studies [35,36,53,54,55], it was surprising to find that all host plants harbored strains of a single species, *B. elkanii*. Although it is possible that other rhizobia types could have existed in untested nodules, the likelihood of high diversity among these seems rare in light of our results. Given that numerous species of *Bradyrhizobium* were identified in the rhizosphere, these results suggest the selection of particular symbionts by the host plants at this site, which may indicate that genotype x genotype interactions are important in this system too. The genetic diversity measures of nodulating rhizobia obtained by [36] in their study locations nearby the location of this study (e.g., H = 9 and Hd = 0.83–0.93) are very similar to our findings (H = 6–9 and Hd = 0.83–0.86) but they still found multiple species of nodulating rhizobia. Furthermore, genetic diversity analyses of the host plants in this study revealed less genetic variation among plants (Table 1), which suggests that they are likely to prefer the same type of rhizobia to associate with those already supported by selection of the same type of nodulating rhizobia by all studied plants. This finding also supports our hypothesis that plants establish symbioses with only a subset of available soil bacteria in their nodules, and confirms several other studies showing that some microbial species typically associate with only a few or a single plant species. This fact is well known for pathogens as well as beneficial interactions, e.g., in the legume–rhizobium relationship. For example, *Sinorhizobium meliloti* effectively colonizes *Medicago*, *Melilotus* and *Trigonella*, whereas *Rhizobium leguminosarum* induces nodules in *Pisum vicea*, *Lens* and *Lathyrus* plants [56]. However, there are also examples of rhizobia with wide host ranges [57,58].

Selection of only one species by the host plants may reduce the SGS pattern of kinship in nodulating rhizobia. A non-significant Mantel test also supports this idea by showing that genetic distance of isolates is not associated with geographic distance between pairs of nodules (r = 0, *p* = 0.99, Figure 6). Additionally, plant genetic distances are required to explain a small but significant amount of the observed variation as our stepwise regression, revealing such impacts of the host genotype on the genotype of the nodulating rhizobia (Table 4). Despite the expectation that plant genotype would explain a greater amount of variation in nodulating bacteria, other studies have also found a lack of strong influence by plant hosts on rhizobia diversity. For example, in a study on nodulating rhizobia of *Lotus japonicus* (Regel) K. Larsen, the plant genotype explained only 4.91% of the variation in nodulating rhizobia [24]. The lack of influence of geographic distance in structuring-nodulating rhizobia is in line with results from ref. [36], which were not able to find a significant correlation between geographic and bacterial genetic distances at larger spatial scales either. Their finding is consistent with other studies demonstrating that genetic structure of soil bacteria is largely independent of geographic distance [59]. Results from my study show that plant genetic distance explains only about 1.2% variation in diversity of nodulating rhizobia, while, by adding geographic distance, the new model explains 1.6% of variation in nodulating rhizobia diversity. It is likely that on small scales, host plant and the geographic distance between pairs of hosts, cumulatively, control take up of specific rhizobia in the soil to associate with the host plant.

Along with geographic distance, soil influence on bacterial communities has been investigated in several studies, and ref. [24] observed that while *Bradyrhizobium* were the major rhizobia symbionts of cowpea, irrespective of plant genotype and soil type, the presence of other genera including *Enterobacter*, *Chryseobacterium*, and *Sphingobacterium* was significantly correlated with the soil type and, to a lesser extent, plant genotype. However, ref. [37] indicated that for rhizobia–host interaction, the plant genotype has a higher influence on the selection of the bacterial symbiont than soil type, due to the fact that the abundance of major OTUs differed in their study under the influence of plant genotype. In ref. [60], it was proposed that the bulk soil surrounding the rhizosphere might introduce low levels of restrictions and/or promote the recruitment of a subset of bacteria that colonize the rhizosphere. Although it might be helpful to analyze the rhizosphere for chemical properties to gain a comprehensive understanding of the factors influencing the microbial communities in the nodules, we postulate that in our fine scale study the soil matrix is quite homogeneous and the role of environmental influences would have been minimized given the very small study area. Congruently, there is no general decision about the key player, which means that both factors can dominate depending on biotic and abiotic conditions [1], and there are several contrasting reports recognizing plant or soil type as the dominant factor [61,62,63]. Although soil type and pH appear to influence free-living soil bacterial communities [17], ref. [64] found that rhizobia housed in nodules of different species of wild *Lotus* were a subset of those in the surrounding soil, indicating a strong role for plant host to choose particular rhizobia genotypes.

## 4. Materials and Methods

### 4.1. Plant and Rhizobia Collection

**Figure 9:**
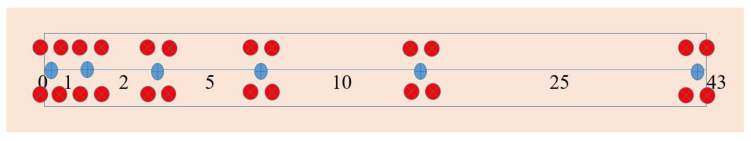
Diagram of one sampling plot. Blue points represent the sampling point at distance intervals of 0, 1, 2, 5, 10, and 25 m from the previous point. At each distance interval, two plants (red points) each were sampled in opposite directions 0.5-m perpendicular to a central point (blue dot), for a total of 24 plants sampled per plot.

In Oktibbeha Co, Mississippi, June 2017, 70 plants were sampled in three consecutive linear plots starting at 33.35939, −88.86587, with 200 m distance between the first and the second plots, 55 m distance between the second and third plots, and with a total length of about 400 m for all three studied plots. Within each plot, plants were sampled at distance intervals of 0, 1, 2, 5, 10, and 25 m from the previous point. At each distance interval, two plants each were sampled in opposite directions 0.5 m perpendicular to a central point, for a total of 24 plants sampled per plot (Figure 9). Plot two included 22 plants as two plants could not be identified at one of the points at distance 10 m. The two sampled plants at each point were within ca. 12 cm of one another (Figure 9). Whole plants, including roots, were carefully excavated from the soil. Plants were stored separately in plastic bags on ice in the field. Plants were kept at 4 °C until DNA could be extracted. All nodules were removed from the roots and stored at 4 °C until they were plated on growth medium.

Leaf samples to be used in DNA extraction were kept on ice in the field and then stored in silica gel or at −80 °C prior to DNA extraction. Genomic DNA was extracted using a CTAB method [65]. Each sampled plant was genotyped at 14 trinucleotide microsatellite loci (Table 5) using a multiplexed genotyping approach to quantify genetic structure. For each sample, three multiplex amplification reactions with five, four, and five loci per reaction respectively, were performed in a final volume of 10 μL in the presence of 10 ng of template DNA, 100 µmole of each of the reverse and tagged fluorescent label primers and 10 µmole of tagged forward primer using a KAPA 2G Fast Multiplex PCR kit (Kapa Bio-systems, Wilmington, Massachusetts). Tag sequences were derived from [66], and the tag in the 5′ forward primer matched the sequence of the fluorescent labeled primer (Table 5). The thermal cycler program used to amplify loci included 3 min at 95 °C, 30 cycles of 15 s at 95 °C, 30 s at 60 °C, and 30 s at 72 °C, and a final extension step of 1 min at 72 °C. Successful amplification of the samples was checked by agarose gel electrophoresis, and amplified products were genotyped at the Arizona State University DNA Lab with LIZ 600 size standard. Individual alleles were sized using GeneMarker software (SoftGenetics, State College, Pennsylvania).

One to two nodules per plant were surface sterilized with 1% hypochlorite for 5 min then washed in sterile water. Later, they were placed in 70% ethanol for 5 min and then were washed three times with sterile distilled water each for 1 min. After surface sterilization, nodules were ground to release rhizobia, and this mixture was plated on solid agar MAG medium [67] in petri dishes at 30 °C until colonies appeared, ca. 4–10 days after plating. One colony per sample that could be morphologically identified as *Bradyrhizobium* (i.e., creamy yellow color, smooth margins, medium sized, and round appearance), following [68,69], was randomly picked and suspended in 50 μL 1× TE buffer solution (pH = 8). Even though there are some studies suggesting the possibility of multiple strains per nodule (e.g., [70]), we selected only one colony per sample because only a single strain of rhizobia is typically found in a nodule as it arises from infection by a single bacterial cell (e.g., [71,72]). Prior to their use directly in PCR, the cells were lysed by heating at 65 °C for 5 min. *TruA*, a housekeeping gene involved in translation and ribosomal biogenesis [73] and that is capable of distinguishing *Bradyrhizobium* strains [36,48,74] was used to characterize nodulating rhizobia. Bacterial samples were amplified with *truAB*-F/R primers specific for *Bradyrhizobium* [48] and then sequenced using Sanger sequencing. PCR was used to amplify the region in 12.5 μL volume, containing 1 μL DNA, 1× LongAmp buffer (New England Biolabs, Ipswich, MA, USA), 1.5 U LongAmp Taq (New England Biolabs), 0.32 mM dNTP’s, 0.4 μM forward primer, and 0.4 μM reverse primer (Integrated DNA Technologies, Coralville, IA, USA). The thermal cycler program consisted of heating to 95 °C for 5 min, followed by 11 cycles of 94 °C for 45 s, 60 °C for 1 min. decreased by 1.0 °C per cycle, 72 °C for 1 min., 26 cycles of 94 °C for 45 s, 50 °C for 1 min., 72 °C for 1 min., and an elongation step of 72 °C for 10 min. Amplified products were subjected to agarose gel electrophoresis to check for the presence of a single band of the expected size for *truA*. Successfully amplified products (one sequence per sample) were cleaned by adding 0.2x Antarctic Phosphatase buffer, 5 units of Exonuclease I, and 1.25 units of Antarctic Phosphatase (New England Biolabs, Ipswich, MA, USA), to 7 μL of PCR product. This mixture was heated to 37 °C for 15 min followed by 80 °C for 15 min. Once the samples were cleaned, cycle sequencing was conducted in 10 μL reactions using either the forward or reverse primer and Big Dye version 3.1 (Life Technologies, Carlsbad, CA, USA). Forward and reverse primer sequences were generated for all individuals using the PCR primers. Sequenced samples were dried and sent to the Arizona State University DNA Lab for capillary electrophoresis. Rhizobia forward and reverse sequences were edited and assembled into a consensus sequence for each sample using Sequencher version 4.7 (Gene Codes Corporation, Ann Arbor, MI, USA). Sequences were aligned using the alignment algorithm in Geneious v.10.2.5 (Biomatters, Inc. Newark, NJ, USA).

For bacterial community sequencing of rhizosphere samples, whole-community DNA was extracted from each rhizosphere sample (*n* = 70 plants) using 0.5 g of soil and the FastDNA™ SPIN Kit for soil isolation (MP Biomedicals, Solon, OH, USA). We were unsuccessful in getting *truA* to amplify consistently in these samples. Thus, we used the 16S rRNA gene to characterize rhizosphere rhizobia communities because this gene has been broadly used in prokaryote taxonomy and phylogenetic reconstructions and sequences are widely available across prokaryotic lineages [75]. Besides, previous studies have shown that assessment of rhizobial genotypic diversity relevant to ecologically oriented studies can be achieved by 16S rRNA sequencing [76]. Using primers 319F and 806R designed for the V3 and V4 region of the 16S rRNA gene [77], amplicons were produced using a 2-step PCR method [77] and sequenced on an Illumina (San Diego, CA, USA) instrument. Each step 1 PCR reaction contained 1× Phusion Taq master mix (New England Biolabs, Ipswich, MA, USA), primers 319F and 806R (0.4 µM each), 3% DMSO, and 5 ng genomic DNA. The thermal cycler program included the following cycles: an initial denaturation at 94 °C for 3 min, 20 cycles of denaturation at 94 °C for 30 s, annealing at 58 °C for 30 s, elongation at 72 °C for 1 min, and a final elongation step at 72 °C for 7 min. Successful amplifications were tested by running a small amount using agarose gel electrophoresis. The second PCR, which added barcodes and adapters, and remaining steps in library preparation were conducted according to [77] at the Microbiome Service Lab at the University of Maryland School of Medicine. All samples were sequenced together in a single run on a MiSeq instrument (Illumina, San Diego, CA, USA) using paired end reads and length of 300 bp each.

To identify bacterial species in the rhizosphere samples, bioinformatics analysis and annotation of the output data were carried out following Berlanas et al. (2019) using QIIME2 [78]. This software provides quality filtering, picking operational taxonomic units (OTUs), taxonomic assignment, phylogenetic reconstruction, diversity analysis, and graphical displays [79]. Sequences were demultiplexed by sample at the Microbiome Service Lab at the University of Maryland School of Medicine using a dual-barcode strategy, a mapping file linking barcode to samples, and QIIME-dependent script of split_libraries.py and split_sequence_file_on_sample_ids.py, [77]. Primer sequences were removed from each read using q2-cutadapt plugin, and sequence quality control and feature table construction were conducted using a DADA2 pipeline [80]. Chimeras for combined runs were removed per the DADA2 pipeline. Amplicon sequence variants (ASVs) generated by DADA2 were taxonomically classified using the scikit-learn classifier [81] trained with the SILVA v132 16S rRNA gene sequence database [82]. After taxonomic classification, sequences belonging to Rhizobiales, which includes Bradyrhizobiaceae, were extracted from the dataset.

### 4.2. Data Analysis

The plant dataset was tested for presence of unique multilocus genotypes using the Poppr package [83] in RStudio statistical software v. 1.1.456 [84]. Genetic diversity of plants was assessed as mean number of alleles per locus (A), percent of polymorphic loci (P), observed heterozygosity (H_O_), expected heterozygosity (H_E_), and inbreeding coefficient (F_IS_) using GenAlEx version 6.503 [85]. For each locus, departure from Hardy–Weinberg expectations was tested through permutations of alleles among individuals and statistical significance was assessed using a *p*-value of 0.001, which is adjusted using the sequential Bonferroni correction for multiple comparisons [86]. A Principal Coordinates Analysis (PCoA) was conducted using GenAlEx version 6.503 [85] to explore dissimilarities among samples in the three studied plots. We performed analysis of spatial genetic structure (SGS) for plant samples using SPAGeDi 1.5 [87]. The plant microsatellite loci were analyzed in each pair of individuals using pairwise relatedness coefficients according to Ritland’s estimator (Equation (5) in [88]), which has been shown to be the best estimator, especially with highly polymorphic markers [43]. Seventeen distance intervals, from 1 to 384 m, were plotted to maximize the number of pairwise comparisons. Spatial genetic structure was tested by assessing the significance of the regression slope (b_F_) of pairwise statistics (F_ij_) on ln (distance) using 9999 randomizations of the individual spatial positions and obtaining 95% confidence intervals (CIs) after bootstrapping (1000 iterations) using SPAGeDi 1.5 [87]. Spatial genetic structure was also quantified by the “Sp” statistic, which estimates SGS intensity and is calculated as − b_F_/(1 − F_(1)_), where F_(1)_ is the mean F_(ij)_ between individuals belonging to the first distance interval and b_F_ is the regression slope of F_(ij)_ on r_ij_ (physical distance between samples i and j) [43]. F_(1)_ can be considered a good estimate of the kinship between pairs of neighbors, on the condition that the first distance interval contains enough pairs of individuals to obtain reasonably precise F_(1)_ values [43]. Standard errors for the estimates of the kinship coefficients per distance class were estimated using a jackknife procedure over the loci. Then, values of the average genetic relatedness statistic between pairs of individuals separated by given distance intervals were plotted using a spatial autocorrelogram.

The sequence of *truA* from each nodule was checked against GenBank sequences via BLAST-n searches [89] to identify closely matched bacterial strains. Rhizobia *truA* sequences were aligned in Geneious v.10.2.5 (Biomatters, Inc. Newark, NJ, USA) using the internal alignment algorithm and compared against five reference sequences of taxonomically valid *Bradyrhizobium* retrieved from GenBank [89] in a phylogenetic tree. The reference sequences included *B. canariense* BTA1 (Accession: JX064276), *B. elkanii* USDA 76 (Accession: JX064277), *B. japonicum* USDA 6 (Accession: JX064272), *B. liaoningense* USDA 3622 (Accession: JX064278), and *B. yuanmingense* CCBAU 10,071 (Accession: JX064271). These five strains are informative for identifying *Bradyrhzobium* species which associate with plants from Fabaceae [36,53]. We used jModeltest2 [90,91] to select HKY+G [92] as the best fitting model of molecular evolution according to the BIC MrBayes v. 3.2.3 [93] was used for phylogenetic reconstruction in the Cipres Science Gateway [94]. We conducted Markov Chain Monte Carlo (MCMC) 5 million generations, sampling every 1000 points. Prior to determining the posterior probability of the trees with the highest likelihood, 1250 trees were discarded as burn-in. A consensus tree is reported with posterior probability indicating support for clades. Sequence diversity analysis including number of haplotypes (H), haplotype diversity (Hd), and π for each plot and the entire dataset was conducted using DnaSP v5 [95].

Analysis of spatial genetic structure (SGS) of nodulating rhizobia was conducted for each plot separately and all plots combined using SPAGeDi 1.5 [87]. Rhizobia *truA* haplotype sequences were converted to SPAGeDi 1.5 input using haplotype codes generated by SPADS 1.0 [96]. All settings in SPAGeDi 1.5 were the same as those used in analysis of SGS for the plants. Spatial genetic structure was tested by assessing the significance of the regression slope (b_F_) of pairwise statistics (Fij) on ln (distance) using 9999 randomizations of the individual spatial positions and obtaining 95% confidence intervals (CIs) after bootstrapping (1000 iterations) [87], and seventeen distance intervals, from 1 to 384 m, were plotted to maximize the number of pairwise comparisons.

Isolation-by-distance for the plant hosts and nodulating rhizobia was investigated separately by comparing pairwise population genetic and geographic distances in a Mantel test [97]. Pairwise genetic distances for plant individuals were calculated using GenAlEx [85], and for rhizobia *truA* sequences using Mega v.6 [98]. Geographic distances between collected individuals were calculated using a modification of the haversine formula [99] based on the coordinates of the sampled plants in GenAlEx [85]. The Mantel test was performed using PASSaGE 2 [100], and 1000 permutations of the datasets were used to assess significance of the correlation. A stepwise regression analysis using IBM SPSS Statistics v.25 [101] was also performed to test whether plant genetic and physical distance can be considered as explanatory variables for rhizobia genetic distances in the nodules.

Sequences of Rhizobiales 16S rRNA that were generated from rhizosphere samples were compared against sequences in GenBank [89] through BLASTn searches to identify closely matching sequences. The highest scoring, as determined by e-value, sequence identified to the species level was selected as the best matching sequence. The relative proportions of each identified strain across all plots were compared to have an understanding of the diversity and abundance of potential symbionts for *C. fasciculata* in the rhizospheric soil.

## 5. Conclusions

No study to date has been conducted on genetic diversity and structure of legume rhizobia system within *Chamaecrista fasciculata* and at the fine scale studied here. Here we showed that there is fine-scale genetic structure in the host plant and its nodulating rhizobia, and that plant genotype along with geographic distance contribute minimally to explaining genetic distance among nodulating rhizobia. The data indicate that co-located host plant individuals are likely to be more genetically similar than those more distant probably because large seeds are maintained close to the maternal plant. The low dispersal ability of bacteria in soil and specificity between legume and rhizobia species may explain the common pattern of structure between rhizobia and their host plants. Nevertheless, the small amount of variation in nodulating rhizobia genetic distances explained by host genetic distance and geographic distance suggests that there may be other environmental factors influencing these interactions. We also found that the only rhizobia strain retrieved from sampled host plant nodules belonged to *Bradyrhizobium elkanii*, which was also the most common form in the rhizosphere. This finding reveals a relatively high degree of plant selection among available rhizobia in soil. To understand to what degree this strain-specific legume rhizobia symbioses can develop, further studies in particular habitats are needed.

## Figures and Tables

**Table 1:** Genetic diversity of the host plant, *Chamaecrista fasciculata*.

Plot	N	A	P	H_O_	H_E_	F_IS_	Significant Deviation from HWE
1	24	5.1	100	0.48	0.55	0.09	no loci
2	22	4.35	100	0.49	0.52	0.1	1 locus (*p* = 0.001)
3	24	5	100	0.48	0.56	0.13	1 locus (*p* = 0.001)
All	70	6.78	100	0.49	0.58	0.17	3 loci (*p* = 0.001)

N = number of individuals sampled; A = mean number of alleles per locus; P = percentage of polymorphic loci; H_O_ = observed heterozygosity; H_E_ = expected heterozygosity; F_IS_ = inbreeding coefficient; HWE = Hardy–Weinberg equilibrium.

**Table 2:** Fine-scale genetic structure in *Chamecrista fasciculata* host plants and nodulating *Bradyrhizobium elkanii* in the studied plots.

Plot	Distance Range (m)	b_F_	*p*-Value	F_1_	Sp Statistic
**Plant**					
Plot 1	1–43	−0.0138	0.001 *	0.0073	0.0139
Plot 2	1–43	−0.0063	0.125	−0.0165	0.0062
Plot 3	1–43	−0.0049	0.145	−0.0068	0.0048
Plots 1–3	1–384	−0.0121	0.000 *	0.0451	0.0127
**Rhizobia**					
Plot 1	1–43	−0.0165	0.046 *	−0.0212	0.0162
Plot 2	1–43	0.0172	0.075	−0.0583	−0.0162
Plot 3	1–43	−0.0009	0.882	−0.008	0.0009
Plots 1–3	1–384	−0.0031	0.041 *	0.0038	0.0031

b = the regression slope of multilocus kinship coefficients for pairs of individuals against the logarithm of geographic distance separating members of the pair; *p*-value = level of significance; F1 = the mean kinship coefficient (Fij) between individuals belonging to the first distance interval; Sp statistics = −bF /(1 − F1); CI = confidence interval. Note: Star shows significant values.

**Table 3:** Genetic diversity of nodulating *Bradyrhizobium elkanii* in the *truA* gene across the studied plots.

Plot	N	H	Hd	π
1	19	8	0.86	0.04175
2	15	6	0.829	0.03457
3	19	9	0.86	0.03679
Combined (1–3)	53	6	0.730	0.03338

N = number of sequences; H = number of haplotypes; Hd = haplotype diversity; π = a measure of the average differences between pairs of sequences.

**Table 4:** Stepwise regression assigning plant genetic and geographic distance as predictors of nodulating *Bradyrhizobium elkanii* genetic distances.

Predictor	Adjusted r Square	*p*-Value
Plant genetics, constant	0.012	0.000
Plant genetics, geographic distance, constant	0.016	0.000

**Table 5:** Characteristics of 14 microsatellite loci used to evaluate genetic structure in Chamaecrista fasciculata.

Locus *	Forward and Reverse Sequence (5′-3′)	Multiplex Group	Fluorescent Label **	Allele Size	Repeat Type
Cf1394	F: GAAAAGGCGTCACCAACACC R: CGTCCATGGCTGCTACTGC	1	NED	336–399	(AGA)_8_
Cf17494	F: TTGGGGGATGACAAAAGTGG R: CCTCAAAATCAAAAGATTGAAACG	4	VIC	200–236	(AAG)_7_
Cf3118	F: CCTCAAAATCAAAAGATTGAAACG R: GGTGAAGGCGAAGAAACAGG	1	PET	200–239	(CCA)_6_
Cf3411	F: GACGGCAAAGAATCCAAAGG R: TCAGTGGATCTGCTTTCTCTCC	3	NED	295–319	(CCG)_7_
Cf4935	F: AGGAAGTGTTGATTCTGCAACC R: AGCCCCTTCACACTCAGTCC	4	PET	192–225	(AAC)_5_ … (AAC)_7_
Cf5782	F: CTTCCTCAGGGTCACAGAACC R: AAAATCCGAGAGCCATGACG	3	NED	189–213	(CTT)_6_
Cf6822	F: CCACTACTATCCCTATCAACAACAGC R: CGTTGAGCATCCACATCAGG	1	PET	209–218	(CCA)_6_
Cf6895	F: TTCACGAGGACCCAGTAGGG R: AGAAGGCGAGACCAGAGAGC	1	FAM	203–245	(CAT)_6_
Cf7140	F: GAGAAGGGAGTGGTCCTAATGG R: TGAGAGGCATTTGAGTCTTGC	4	FAM	185–206	(TAG)_8_
Cf8757	F: AGTAGCACCACACCCTCACG R: TTCCTCCAATCCCCTTTTCC	4	FAM	379–433	(ATC)_6_
Cf9980	F: GCTGCTCTGGGAATATCACG R: CTGCGTAGCCACTTCACTCG	1	NED	205–352	(GAA)_7_
Cf10002	F: AGAGAGTGCCCAGGTGAAGG R: GATCCTCGTCGCTCATAGGG	1	VIC	219–246	(TGG)_9_
Cf20956	F: ATTACCAAGAGTTGGAAAATATCG R: CCACCCATTCCAGAGTGTCC	3	FAM	246–300	(ATG)_9_
Cf4487	F: CGAGGAGCCTCTTCTTCAGG R: CTGGGCTCATGTTTCTGAGG	4	NED	190–217	(TCT)_12_

* The locus names correspond to the sequence names in the transcriptome file version_1.fasta available at https://serc.carleton.edu/exploring_genomics/chamaecrista/variation_.html. ** Tag names and sequences (5′-3′) are as following: NED = M13A, TGTAAAACGACGGCCAGT; VIC = T7term, CTAGTTATTGCTCAGCGGT; PET = M13B, CACTGCTTAGAGCGATGC; FAM = M13(−21), TGTAAAACGACGGCCAGT.

## References

[1] Berg G., Smalla K. (2009). Plant species and soil type cooperatively shape the structure and function of microbial communities in the rhizosphere. FEMS Microbiol. Ecol..

[2] Diouf M., Baudoin E., Dieng L., Assigbetsé K., Brauman A. (2010). Legume and gramineous crop residues stimulate distinct soil bacterial populations during early decomposition stages. Can. J. Soil Sci..

[3] Ladygina N., Hedlund K. (2010). Plant species influence microbial diversity and carbon allocation in the rhizosphere. Soil Biol. Biochem..

[4] McLaren J.R., Turkington R. (2011). Plant identity influences decomposition through more than one mechanism. PLoS ONE.

[5] Bever J.D., Dickie I.A., Facelli E., Facelli J.M., Klironomos J., Moora M., Rillig M.C., Stock W.D., Tibbett M., Zobel M. (2010). Rooting theories of plant community ecology in microbial interactions. Trends Ecol. Evol..

[6] Ettema C.H., Wardle D.A. (2002). Spatial soil ecology. Trends Ecol. Evol..

[7] Bouffaud M.L., Poirier M.A., Muller D., Moënne-Loccoz Y. (2014). Root microbiome relates to plant host evolution in maize and other Poaceae. Environ. Microbiol..

[8] O’Malley M.A. (2007). The nineteenth century roots of ‘everything is everywhere. Nat. Rev. Microbiol..

[9] Emmett B., Nelson E.B., Kessler A., Bauerle T.L. (2014). Fine-root system development and susceptibility to pathogen colonization. Planta.

[10] Zhou N., Zhao S., Tian C.Y. (2017). Effect of halotolerant rhizobacteria isolated from halophytes on the growth of sugar beet (*Beta vulgaris* L.) under salt stress. FEMS Microbiol. Lett..

[11] Ehrenfeld J.G., Ravit B., Elgersma K. (2005). Feedback in the plant–soil system. Annu. Rev. Environ. Resour..

[12] Hardoim P.R., van Overbeek L.S., van Elsas J.D. (2008). Properties of bacterial endophytes and their proposed role in plant growth. Trends Microbiol..

[13] Aira M., Monroy F., Domínguez J. (2007). *Eisenia fetida* (Oligochaeta: Lumbricidae) modifies the structure and physiological capabilities of microbial communities improving carbon mineralization during vermicomposting of pig manure. Microb. Ecol..

[14] Zancarini A., Mougel C., Voisin A.S., Prudent M., Salon C., Munier-Jolain N. (2012). Soil nitrogen availability and plant genotype modify the nutrition strategies of *M. truncatula* and the associated rhizosphere microbial communities. PLoS ONE.

[15] Marques J.M., da Silva T.F., Vollu R.E., Blank A.F., Ding G.C., Seldin L., Smalla K. (2014). Plant age and genotype affect the bacterial community composition in the tuber rhizosphere of field-grown sweet potato plants. FEMS Microbiol. Ecol..

[16] Bulgarelli D., Garrido-Oter R., Münch P.C., Weiman A., Dröge J., Pan Y., Mchardy A.C., Schulze-Lefert P. (2015). Structure and function of the bacterial root microbiota in wild and domesticated barley. Cell Host Microbe.

[17] Fierer N., Ladau J. (2012). Predicting microbial distributions in space and time. Nat. Methods.

[18] Vuong H.B., Thrall P.H., Barrett L.G. (2017). Host species and environmental variation can influence rhizobial community composition. J. Ecol..

[19] Pahua V.J., Stokes P.J.N., Hollowell A.C., Regus J.U., Gano-Cohen K.A., Wendlandt C.E., Quides K.W., Lyu J.Y., Sachs J.L. (2018). Fitness variation among host species and the paradox of ineffective rhizobia. J. Evol. Biol..

[20] Heath K.D., Tiffin P. (2007). Context dependence in the coevolution of plant and rhizobial mutualists. Proc. R. Soc..

[21] Rangin C., Brunel B., Cleyet-Marel J.C., Perrineau M.M., Bena G. (2008). Effects of *Medicago truncatula* genetic diversity, rhizobial competition, and strain effectiveness on the diversity of a natural *Sinorhizobium* species community. Appl. Environ. Microbiol..

[22] Crook M.B., Lindsay D.P., Biggs M.B., Bentley J.S., Price J.C., Clement S.C., Clement M.J., Long S.R., Griffitts J.S. (2012). Rhizobial plasmids that cause impaired symbiotic nitrogen fixation and enhanced host invasion. Mol. Plant Microbe Interact..

[23] Kim M., Chen Y., Xi J., Waters C., Chen R., Wang D. (2015). An antimicrobial peptide essential for bacterial survival in the nitrogen-fixing symbiosis. Proc. Natl. Acad. Sci. USA.

[24] Zgadzaj R., Garrido-oter R., Bodker D., Koprivova A., Schulze-lefert P. (2016). Root nodule symbiosis in Lotus japonicus drives the establishment of distinctive rhizosphere, root, and nodule bacterial communities. Proc. Natl. Acad. Sci. USA.

[25] Wang Q., Liu J., Li H., Yang S., Kormoczi P., Kereszt A., Zhu H. (2018). Nodulespecific cysteine-rich peptides negatively regulate nitrogen-fixing symbiosis in a strain-specific manner in *Medicago truncatula*. Mol. Plant Microbe Interact..

[26] Perret X., Staehelin C., Broughton W.J. (2000). Molecular basis of symbiotic promiscuity. Microbiol. Mol. Biol. Rev..

[27] Wang D., Yang S., Tang F., Zhu H. (2012). Symbiosis specificity in the legume: Rhizobial mutualism. Cell Microbiol..

[28] Lu J., Yang F., Wang S., Ma H., Liang J., Chen Y. (2017). Co-existence of rhizobia and diverse non-rhizobial bacteria in the rhizosphere and nodules of *Dalbergia odorifera* seedlings inoculated with B*radyrhizobium elkanii, Rhizobium multihospitium*-like and *Burkholderia pyrrocinia*-like strains. Front. Microbiol..

[29] Irwin H.S., Barneby R.C. (1982). The American Cassinae: A synoptical revision of Leguminosae tribe Cassieae subtribe Cassinae in the New World. Mem. N. Y. Bot..

[30] Reeves D.W., Hatfield J.L., Stewart B.A. (1994). Cover Crops and Rotations. Advances in Soil Science: Crops Residue Management.

[31] Rodríguez-Kábana R., Kokalis-Burelle N., Robertson D.G., Weaver C.F., Wells L. (1995). Effects of Partridge Pea–Peanut rotations on populations of *Meloidogyne arenaria*, incidence of *Sclerotium rolfsii*, and yield of peanut. Nematropica.

[32] Singer S., Doyle J., May G., Cannon S., Maki S., Illut D. Exploring *Chamaecrista Fasciculata* Genomics Data [Online: 2009]. http://serc.carleton.edu/exploring_genomics/chamaecrista/chamaecrista_tr.html.

[33] Parker M. (1999). Mutualism in metapopulations of legumes and rhizobia. Am. Nat..

[34] Parker M., Kennedy D.A. (2006). Diversity and relationships of *Bradyrhizobium* from legumes native to eastern North America. Can. J. Microbiol..

[35] Andrews M., Andrews M.E. (2017). Specificity in legume–rhizobia symbioses. Int. J. Mol. Sci..

[36] Dorman H., Wallace L. (2019). Diversity of nitrogen-fixing symbionts of *Chamaecrista fasciculata* (Partridge Pea) across variable soils. Southeast. Nat..

[37] Leite J., Fischer D., Rouws L.F., Fernandes-Júnior P.I., Hofmann A., Kublik S., Schloter M., Xavier G.R., Radl V. (2017). Cowpea nodules harbor non-rhizobial bacterial communities that are shaped by soil type rather than plant genotype. Front. Plant Sci..

[38] Jiang Y., Li S., Li R., Zhang J., Liu Y., Lv L., Zhu H., Wu W., Li W. (2017). Plant cultivars imprint the rhizosphere bacterial community composition and association networks. Soil Biol. Biochem..

[39] Igolkina A., Bazykin G.A., Chizhevskaya E.P., Provorov N.A., Andronov E.E. (2018). The Evolutionary Moulding in plant-microbial symbiosis: Matching population diversity of rhizobial nodA and legume NFR5 genes. bioRxiv.

[40] Portnoy S., Willson. M.F. (1993). Seed dispersal curves: Behavior of the tail of the distribution. Evol. Ecol..

[41] Willson M.F. (1993). Dispersal mode, seed shadows, and colonization patterns. Vegetatio.

[42] Schupp E.W., Fuentes M. (1995). Spatial patterns of seed dispersal and the unification of plant population ecology. Écoscience.

[43] Vekemans X., Hardy O.J. (2004). New insights from fine-scale spatial genetic structure analyses in plant populations. Mol. Ecol..

[44] Loiselle B.A., Sork V.L., Nason J., Graham C. (1995). Spatial genetic structure of a tropical understory shrub, *Psychotria officinalis* (Rubiaceae). Am. J. Bot..

[45] Rousset F. (2000). Genetic differentiation between individuals. J. Evol. Biol..

[46] Hardy O.J. (2003). Estimation of pairwise relatedness between individuals and characterization of isolation-by-distance processes using dominant genetic markers. Mol. Ecol..

[47] Fenster C.B., Vekemans X., Hardy O.J. (2003). Quantifying gene flow from spatial genetic structure data in a metapopulation of *Chamaecrista fasciculata* (Leguminosae). Evolution.

[48] Vinues Y.M., Tian C.T., Sui X.H., Chen W.F., Chen W.X. (2012). Robust markers reflecting phylogeny and taxonomy of rhizobia. PLoS ONE.

[49] Van Cauwenberghe J., Verstraete B., Lemaire B., Lievens B., Michiels J., Honnay O. (2014). Population structure of root nodulating *Rhizobium leguminosarum* in *Vicia cracca* populations at local to regional geographic scales. Syst. Appl. Microbiol..

[50] Bjornsgaard Aas A., Andrew C.J., Blaalid R., Vik U., Kauserud H., Davey M.L. (2019). Fine-scale diversity patterns in belowground microbial communities are consistent across kingdoms. FEMS Microbiol. Ecol..

[51] Klock M.M., Barrett L.G., Thrall P.H., Harms K.E. (2015). Host-promiscuity in symbiont associations can influence exotic legume establishment and colonization of novel ranges. Divers. Distrib..

[52] Ndlovu J., Richardson D.M., Wilson J.R.U., Le Roux J.J. (2013). Co-invasion of South African ecosystems by an Australian legume and its rhizobial symbionts. J. Biogeogr..

[53] Koppell J.H., Parker M.A. (2012). Phylogenetic clustering of *Bradyrhizobium* symbionts on legumes indigenous to North America. Microbiology.

[54] Parker M.A. (2015). The spread of *Bradyrhizobium* lineages across host legume clades: From Abarema to Zygia. Microb. Ecol..

[55] Santos J.M., Casaes Alves P.A., Silva V.C., Kruschewsky Rhem M.F., James E.K., Gross E. (2017). Diverse genotypes of *Bradyrhizobium* nodulate herbaceous *Chamaecrista* (moench) (Fabaceae, caesalpinioideae) species in Brazil. Syst. Appl. Microbiol..

[56] Bais H.P., Weir T.L., Perry L.G., Gilroy S., Vivanco J.M. (2006). The role of root exudates in rhizosphere interactions with plants and other organisms. Annu. Rev. Plant Biol..

[57] Schmeisser C., Liesegang H., Krysciak D., Krysciak D., Bakkou N., Le Quéré A., Wollherr A., Henemeyer I., Mogenstern B., Pommerening A. (2009). Rhizobium sp. strain NGR234 possesses a remarkable number of secretion systems. Appl. Environ. Microbiol..

[58] Safronova V., Belimov A., Sazanova A., Chirak E., Kuznetsova I., Andronov E., Pinavea A., Tsyganova A., Seliverstova E., Kitaeva A. (2019). Two broad host range rhizobial strains isolated from relict legumes have various complementary effects on symbiotic parameters of co-inoculated plants. Front. Microbiol..

[59] Fierer N., Jackson R.B. (2006). The diversity and biogeography of soil bacterial communities. Proc. Natl. Acad. Sci. USA.

[60] Fernández-Gómez B., Maldonado J., Mandakovic D., Gaete A., Gutiérrez R.A., Maass A., Cambiazo V., González M. (2019). Bacterial communities associated to Chilean altiplanic native plants from the Andean grassland’s soils. Sci. Rep..

[61] Grayston S.J., Wang S., Campbell C.D., Edwards A.C. (1998). Selective influence of plant species on microbial diversity in the rhizosphere. Soil Biol. Biochem..

[62] Girvan M.S., Bullimore J., Pretty J.N., Osborn A.M., Ball A.S. (2003). Soil type is the primary determinant of the composition of total and active bacterial communities in arable soils. Appl. Environ. Microbiol..

[63] Nunan N., Daniell T.J., Singh B.K., Papert A., Mc Nicol J.W., Prosser J.I. (2005). Links between plant and rhizoplane bacterial communities in grassland soils, characterized using molecular techniques. Appl. Environ. Microbiol..

[64] Sachs J.L., Kembel S.W., Lau A.H., Simms E.L. (2009). In situ phylogenetic structure and diversity of wild *Bradyrhizobium* communities. Appl. Environ. Microbiol..

[65] Dellaporta S.L., Wood J., Hicks J.B. (1983). A plant DNA mini preparation: Version II. Plant Mol. Biol. Rep..

[66] Culley T.M., Stamper T.I., Stokes R.L., Brzyski J.R., Hardiman N.A., Klooster M.R., Merritt B.J. (2013). An efficient technique for primer development and application that integrates fluorescent labeling and multiplex PCR. Appl. Plant Sci..

[67] Castillo F. (2014). Evaluation of Nitrogen Needs and Efficiency of Rizhobia Strains to Provide Nitrogen to Chipilin (*Crotalaria Longirostrata* HOOK. AND ARN). Master’s Thesis.

[68] Sylvester-Bradley R., Thornton P., Jones P. (1988). Colony dimorphism in *Bradyrhizobium* strains. Appl. Environ. Microbiol..

[69] Fuhrmann J.J. (1990). Symbiotic effectiveness of indigenous soybean *Bradyrhizobia* as related to serological, morphological, rhizobiotoxine, and hydrogenase phenotypes. Appl. Environ. Microbiol..

[70] Checcucci A., Azzarello E., Bazzicalupo M., Galardini M., Lagomarsino A., Mancuso S., Marti L., Marzano M.C., Mocali S., Squartini A. (2016). Mixed Nodule Infection in Sinorhizobium meliloti–Medicago sativa Symbiosis Suggest the Presence of Cheating Behavior. Front. Plant Sci..

[71] Denison R.F., Kiers E.T. (2004). Why are most rhizobia beneficial to their plant hosts, rather than parasitic?. Microbes Infect..

[72] Simms E.L., Taylor D.L., Povich J., Shefferson R.P., Sachs J., Urbina M., Tausczik Y. (2006). An empirical test of partner choice mechanisms in a wild legume-rhizobium interaction. Proc. R. Soc. B Biol. Sci..

[73] Ahn K.S., Ha U., Jia J., Wu D., Jin S. (2004). The *truA* gene of Pseudomonas aeruginosa is required for the expression of type III secretory genes. Microbiology.

[74] Vinuesa P., Silva C., Werner D., Martínez-Romero E. (2005). Population genetics and phylogenetic inference in bacterial molecular systematics: The roles of migration and recombination in *Bradyrhizobium* species cohesion and delineation. Mol. Phylogenet. Evol..

[75] Lapage S.P., Sneath P.H.A., Lessel E.F., Skerman V.B.D., Seeliger H.P.R., Clark W.A. (1992). Chapter 3, Rules of Nomenclature with Recommendations. International Code of Nomenclature of Bacteria.

[76] Fox G.E., Wisotzkey J.D., Jurtshuk P. (1992). How close is close: 16SrRNA sequence identity may not be sufficient to guarantee species identity. Int. J. Syst. Bacteriol..

[77] Holm J.B., Humphrys M.S., Robinson C.K., Settles M.L., Ott S., Fu L., Yang H., Gajer P., He X., McComb E. (2019). Ultrahigh-throughput multiplexing and sequencing of >500-basepair amplicon regions on the Illumina HiSeq 2500 platform. mSystems.

[78] Bolyen E., Rideout J.R., Dillon M.R., Bokulich N.A., Abnet C.C., Al-Ghalith G.A., Alexander H., Alm E.J., Arumugam M., Asnicar F. (2019). Reproducible, interactive, scalable and extensible microbiome data science using QIIME 2. Nat. Biotechnol..

[79] Caporaso J.G., Kuczynski J., Stombaugh J., Bittinger K., Bushman F.D., Costello E.K., Fierer N., Peña A.G., Goodrich J.K., Gordon J.I. (2010). QIIME allows analysis of high-throughput community sequencing data. Nat. Methods.

[80] Callahan B.J., McMurdie P.J., Rosen M.J., Han A.W., Johnson A.J., Holmes S.P. (2016). DADA2: High Resolution Sample Inference from Amplicon Data. Nat. Methods.

[81] Pedregosa F., Varoquaux G., Gramfort A., Michel V., Thirion B., Grisel O. (2011). Scikit-learn: Machine learning in python. J. Mach. Learn. Res..

[82] Quast C., Pruesse E., Yilmaz P., Gerken J., Schweer T., Yarza P., Peplies J., Glöckner F.O. (2012). The SILVA ribosomal RNA gene database project: Improved data processing and web-based tools. Nucleic Acids Res..

[83] Kamvar Z.N., Tabima J.F., Grünwald N.J. (2014). Poppr: An R package for genetic analysis of populations with clonal, partially clonal, and/or sexual reproduction. PeerJ.

[84] RStudio Team RStudio: Integrated Development Environment for R, Version 1.1.456. Boston, Massachussete, USA. http://www.rstudio.com.

[85] Peakall R., Smouse P.E. (2012). GenAlEx 6.5: Genetic analysis in Excel. Population genetic software for teaching and research-an update. Bioinformatics.

[86] Holm S. (1979). A simple sequentially rejective multiple test procedure. Scand. J. Stat..

[87] Hardy O.J., Vekemans X. (2002). SPAGeDi: A versatile computer program to analyze spatial genetic structure at the individual or population levels. Mol. Ecol. Notes.

[88] Ritland K. (1996). Estimators for pairwise relatedness and inbreeding coefficients. Genet. Res..

[89] National Center for Biotechnology Information (NCBI) National Library of Medicine (US), National Center for Biotechnology Information; Bethesda, MD. https://www.ncbi.nlm.nih.gov/.

[90] Darriba D., Taboada G.L., Doallo R., Posada D. (2012). jModelTest 2: More models, new heuristics, and parallel computing. Nat. Methods.

[91] Guindon S., Gascuel O. (2003). A simple, fast, and accurate method to estimate large phylogenies by maximum likelihood. Syst. Biol..

[92] Hasegawa M., Kishino K., Yano T. (1985). Dating the human–ape splitting by a molecular clock of mitochondrial DNA. J. Mol. Evol..

[93] Ronquist F., Teslenko M., Van Der Mark P., Ayres D.L., Darling A., Höhna S., Larget B., Liu L., Suchard M.A., Huelsenbeck J.P. (2012). MrBayes 3.2: Efficient Bayesian phylogenetic inference and model choice across a large model space. Syst. Biol..

[94] Miller M.A., Pfeiffer W., Schwartz T. (2010). Creating the CIPRES Science Gateway for inference of large phylogenetic trees. Institute of Electrical and Electronics Engineers.

[95] Librado P., Rozas (2009). J. DnaSP v5: A software for comprehensive analysis of DNA polymorphism data. Bioinformatics.

[96] Dellicour S., Mardulyn P. (2014). SPADS 1.0: A toolbox to perform spatial analyses on DNA sequence datasets. Mol. Ecol. Resour..

[97] Mantel N. (1967). The detection of disease clustering and a generalized regression approach. Cancer Res..

[98] Tamura K., Stecher G., Peterson D., Filipski A., Kumar S. (2013). MEGA6: Molecular evolutionary-genetics analysis version 6.0. Mol. Biol. Evol..

[99] Sinnott R.W. (1984). Virtues of the Haversine. Sky Telesc..

[100] Rosenberg M.S., Anderson C.D. (2011). PASSaGE: Pattern Analysis, Spatial Statistics and Geographic Exegesis. Version 2. Methods Ecol. Evol..

[101] IBM Corp (2017). IBM SPSS Statistics for Winodows, Version 25.

